# The coming decade of digital brain research: A vision for neuroscience at the intersection of technology and computing

**DOI:** 10.1162/imag_a_00137

**Published:** 2024-04-18

**Authors:** Katrin Amunts, Markus Axer, Swati Banerjee, Lise Bitsch, Jan G. Bjaalie, Philipp Brauner, Andrea Brovelli, Navona Calarco, Marcel Carrere, Svenja Caspers, Christine J. Charvet, Sven Cichon, Roshan Cools, Irene Costantini, Egidio Ugo D’Angelo, Giulia De Bonis, Gustavo Deco, Javier DeFelipe, Alain Destexhe, Timo Dickscheid, Markus Diesmann, Emrah Düzel, Simon B. Eickhoff, Gaute Einevoll, Damian Eke, Andreas K. Engel, Alan C. Evans, Kathinka Evers, Nataliia Fedorchenko, Stephanie J. Forkel, Jan Fousek, Angela D. Friederici, Karl Friston, Stephen Furber, Liesbet Geris, Rainer Goebel, Onur Güntürkün, Aini Ismafairus Abd Hamid, Christina Herold, Claus C. Hilgetag, Sabine M. Hölter, Yannis Ioannidis, Viktor Jirsa, Sriranga Kashyap, Burkhard S. Kasper, Alban de Kerchove d’Exaerde, Roxana Kooijmans, István Koren, Jeanette Hellgren Kotaleski, Gregory Kiar, Wouter Klijn, Lars Klüver, Alois C. Knoll, Zeljka Krsnik, Julia Kämpfer, Matthew E Larkum, Marja-Leena Linne, Thomas Lippert, Jafri Malin Abdullah, Paola Di Maio, Neville Magielse, Pierre Maquet, Anna Letizia Allegra Mascaro, Daniele Marinazzo, Jorge Mejias, Andreas Meyer-Lindenberg, Michele Migliore, Judith Michael, Yannick Morel, Fabrice O. Morin, Lars Muckli, Guy Nagels, Lena Oden, Nicola Palomero-Gallagher, Fanis Panagiotaropoulos, Pier Stanislao Paolucci, Cyriel Pennartz, Liesbet M. Peeters, Spase Petkoski, Nicolai Petkov, Lucy S. Petro, Mihai A. Petrovici, Giovanni Pezzulo, Pieter Roelfsema, Laurence Ris, Petra Ritter, Kathleen Rockland, Stefan Rotter, Andreas Rowald, Sabine Ruland, Philippe Ryvlin, Arleen Salles, Maria V. Sanchez-Vives, Johannes Schemmel, Walter Senn, Alexandra A. de Sousa, Felix Ströckens, Bertrand Thirion, Kâmil Uludağ, Simo Vanni, Sacha Jennifer van Albada, Wim Vanduffel, Julien Vezoli, Lisa Vincenz-Donnelly, Florian Walter, Laszlo Zaborszky

**Affiliations:** Research Centre, Institute of Neurosciences and Medicine (INM-1), Jülich, Germany; C. & O. Vogt Institute for Brain Research, University Hospital Düsseldorf, Heinrich-Heine University Düsseldorf, Düsseldorf, Germany; Faculty of Mathematics and Natural Sciences, Bergische Universität Wuppertal, Wuppertal, Germany; Université Paris-Saclay, CEA, List, Palaiseau, France; The Danish Board of Technology Foundation, Copenhagen, Denmark; Institute of Basic Medical Sciences, University of Oslo, Oslo, Norway; RWTH Aachen University, Aachen, Germany; Institut de Neurosciences de la Timone, Aix Marseille Université, CNRS, Marseille, France; University of Toronto, Toronto, Canada; Institute of Anatomy I, University Hopsital Düsseldorf, Heinrich Heine University Düsseldorf, Düsseldorf, Germany; College of Veterinary Medicine, Auburn University, Auburn, AL, United States; Department of Biomedicine, University of Basel, Basel, Switzerland; Institute of Medical Genetics and Pathology, University Hospital Basel, Basel, Switzerland; Radboud University Nijmegen, Nijmegen, Netherlands; European Laboratory for Non-Linear Spectroscopy, Department of Biology, University of Florence, Florence, Italy; Neurological Institute Foundation Casimiro Mondino (IRCCS), University of Pavia, Pavia, Italy; Istituto Nazionale di Fisica Nucleare (INFN), Sezione di Roma, Rome, Italy; ICREA, Barcelona, Spain; University Pompeu Fabra, Barcelona, Spain; Laboratorio Cajal de Circuitos Corticales, Centro de Tecnología Biomédica, Universidad Politécnica de Madrid, Madrid, Spain; Instituto Cajal, Consejo Superior de Investigaciones Científicas (CSIC), Madrid, Spain; Paris-Saclay University, Centre National de la Recherche Scientifique, Gif sur Yvette, France; Institute of Computer Science, Heinrich-Heine-Universität Düsseldorf, Düsseldorf, Germany; Institute of Neuroscience and Medicine (INM-6) and Institute for Advanced Simulation (IAS-6) and JARA Institute Brain Structure-Function Relationships (JBI-1/INM-10), Jülich Research Centre, Jülich, Germany; Institute of Cognitive Neurology and Dementia Research, Otto-von-Guericke University Magdeburg, Magdeburg, Germany; German Center for Neurodegenerative Diseases (DZNE), Magdeburg, Germany; Institute of Cognitive Neuroscience, University College London, London, United Kingdom; Institute of Neuroscience and Medicine, Brain and Behaviour (INM-7), Research Centre Jülich, Jülich, Germany; Institute of Systems Neuroscience, Medical Faculty, Heinrich Heine University Düsseldorf, Düsseldorf, Germany; Norwegian University of Life Sciences & University of Oslo, Oslo, Norway; De Montfort University, Leicester, United Kingdom; Department of Neurophysiology and Pathophysiology, University Medical Center Hamburg-Eppendorf, Hamburg, Germany; Montreal Neurological Institute, Montreal, Canada; McGill University, Montreal, Canada; Centre for Research Ethics and Bioethics, Uppsala University, Uppsala, Sweden; Donders Institute for Brain, Behaviour, and Cognition, Radboud University, Nijmegen, Netherlands; Aix Marseille Université, Institut National de la Santé et de la Recherche Médicale, Institut de Neurosciences des Systèmes (INS) UMR1106, Marseille, France; Max Planck Institute for Human Cognitive and Brain Sciences, Leipzig, Germany; University College London, London, United Kingdom; Department of Computer Science, The University of Manchester, Manchester, United Kingdom; Université de Liège, Liège, Belgium; KU Leuven, Leuven, Belgium; VPH Institute, Leuven, Belgium; Maastricht University, Maastricht, The Netherlands; Biopsychology, Institute of Cognitive Neuroscience, Faculty of Psychology, Ruhr-University Bochum, Bochum, Germany; Brain Behaviour Cluster, Department of Neurosciences, School of Medical Sciences, Universiti Sains Malaysia, Kelantan, Malaysia; Institute of Computational Neuroscience, University Medical Center Hamburg-Eppendorf, Hamburg, Germany; Health Sciences Department, Boston University, Boston, MA, United States; Helmholtz Zentrum München, Institut für Entwicklungsgenetik, Neuherberg, Germany; ATHENA Research & Innovation Center, Marousi, Greece; Department of Informatics & Telecom, National and Kapodistrian University of Athens, Zografou, Greece; University Health Network, Toronto, Canada; Epilepsy Center, Department of Neurology, FAU Erlangen-Nuremberg, Erlangen, Germany; Neurophy Lab, ULB Neuroscience Institute, Université Libre de Bruxelles (ULB), Bruxelles, Belgium; Netherlands Institute for Neuroscience (KNAW), Amsterdam, Netherlands; Computational Science and Technology, KTH Royal Institute of Technology, Stockholm, Sweden; Karolinska Institutet (KI), Solna, Sweden; Child Mind Institute, New York, NY, United States; Institute for Advanced Simulation (IAS), Jülich Supercomputing Centre (JSC), Research Centre Jülich, Jülich, Germany; Department of Informatics, Technical University Munich, Munich, Germany; Croatian Institute for Brain Research, School of Medicine, University of Zagreb, Zagreb, Croatia; Humboldt University of Berlin, Berlin, Germany; Faculty of Medicine and Health Technology, Tampere University, Tampere, Finland; Center for Systems, Knowledge Representation and Neuroscience, Ronin Institute, Montclair, NJ, United States; Neuroscience Institute - National Research Council, Rome, Italy; University of Ghent, Ghent, Belgium; Swammerdam Institute for life Sciences, University of Amsterdam, Amsterdam, Netherlands; Department of Psychiatry and Psychotherapy, Central Institute of Mental Health, Heidelberg University, Heidelberg, Germany; Institute of Biophysics, National Research Council, Palermo, Italy; University of Maastricht, Maastricht, Netherlands; University of Glasgow, Glasgow, United Kingdom; University of Brussels, Brussels, Belgium; FernUniversität in Hagen, Faculty of Mathematics and Computer Science, Hagen, Germany; Cognitive Neuroimaging Unit, CEA, DSV/I2BM, INSERM, Universite Paris-Sud, Universite Paris-Saclay, Neurospin Center, Gif/Yvette, France; INFN- the National Institute of Nuclear Physics, Rome, Italy; Biomedical Research Institute & Data Science Institute, Hasselt University, Hasselt, Belgium; Bernoulli Institute for Mathematics, Computer Science and Artificial Intelligence, University of Groningen, Groningen, Netherlands; Kirchhoff-Institut für Physik, Ruprecht-Karls-Universität Heidelberg, Heidelberg, Germany; University of Bern, Bern, Switzerland; Institute of Cognitive Sciences and Technologies - National Research Council, Roma, Italy; Laboratory of Visual Brain Therapy, Sorbonne Université, INSERM, CNRS, Institut de la Vision, Paris, France; Department of Integrative Neurophysiology, VU University, De Boelelaan, Netherlands; Department of Psychiatry, Academic Medical Center, Amsterdam, Netherlands; Université de Mons, Mons, Belgium; Berlin Institute of Health at Charité, Universitätsmedizin Berlin, Berlin, Germany; Charité-Universitätsmedizin Berlin, corporate member of Freie Universität Berlin and Humboldt Universität zu Berlin, Department of Neurology with Experimental Neurology, Berlin, Germany; Bernstein Focus State Dependencies of Learning & Bernstein Center for Computational Neuroscience Berlin, Berlin, Germany; Boston University School of Medicine, Boston, MA, United States; Bernstein Center Freiburg & Faculty of Biology, University of Freiburg, Freiburg im Breisgau, Germany; ProModell Group, Chair for Digital Health, Department of Medical Informatics, Biometry and Epidemiology, Friedrich-Alexander-Universität Erlangen-Nürnberg, Erlangen, Germany; Department of Clinical Neuroscience, Lausanne University Hospital and University of Lausanne, Lausanne, Switzerland; Centre for Research Ethics & Bioethics, Uppsala University, Uppsala, Sweden; Programa de Neuroetica, Centro de Investigationes Filosoficas, Buenos Aires, Argentina; Systems Neuroscience, Institute of Biomedical Investigations August Pi i Sunyer, Barcelona, Spain; Bath Spa University; UKRI ART:AI, University of Bath, Bath, United Kingdom; Allen Institute for Brain Sciences, Seattle, WA, United States; Inria, CEA, Université Paris-Saclay, Palaiseau, France; University of Helsinki, Helsinki, Finland; Institute of Zoology, University of Cologne, Cologne, Germany; Athinoula A. Martinos Center for Biomedical Imaging, Department of Radiology, Massachusetts General Hospital, Boston, MA, United States; Harvard Medical School, Boston, MA, United States; Ernst Strüngmann Institute for Neuroscience in Cooperation with Max Planck Society, Frankfurt, Germany; Inserm, Stem Cell and Brain Research Institute, Université Claude Bernard, Villeurbanne, France; Chair of Robotics, Artificial Intelligence and Real-time Systems, Technical University of Munich, Munich, Germany; Center of Molecular and Behavioral Neuroscience, Rutgers University, Newark, NJ, United States

**Keywords:** human brain, digital research tools, research roadmap, brain models, data sharing, research platforms.

## Abstract

In recent years, brain research has indisputably entered a new epoch, driven by substantial methodological advances and digitally enabled data integration and modelling at multiple scales—from molecules to the whole brain. Major advances are emerging at the intersection of neuroscience with technology and computing. This new science of the brain combines high-quality research, data integration across multiple scales, a new culture of multidisciplinary large-scale collaboration, and translation into applications. As pioneered in Europe’s Human Brain Project (HBP), a systematic approach will be essential for meeting the coming decade’s pressing medical and technological challenges. The aims of this paper are to: develop a concept for the coming decade of digital brain research, discuss this new concept with the research community at large, identify points of convergence, and derive therefrom scientific common goals; provide a scientific framework for the current and future development of EBRAINS, a research infrastructure resulting from the HBP’s work; inform and engage stakeholders, funding organisations and research institutions regarding future digital brain research; identify and address the transformational potential of comprehensive brain models for artificial intelligence, including machine learning and deep learning; outline a collaborative approach that integrates reflection, dialogues, and societal engagement on ethical and societal opportunities and challenges as part of future neuroscience research.

## 1 Introduction

Research in the last decades has yielded impressive progress in our understanding of the human brain. In confronting brain complexity, researchers have studied the brain at different levels of organisation, from the processes at the level of single molecules and genes, synapses, cells, and local circuits to the level of the brain as a whole organ with areas, nuclei, and their networks, involved in a variety of brain functions as well as dysfunction.

Neurological disorders are today the second leading cause of death after heart disease with 276 million DALYS^1^(Disability-Adjusted Life-Years; Global Burden of Disease 2019) (Feigin et al., 2019). In 2010, the total cost of brain disorders in Europe came to €798 billion (Olesen et al., 2012). To address such a challenge, and to develop more effective, causal therapies, we need to better understand the fundamentals of how the brain works. Hereby, we are inevitably confronted with the complexity of the organ and its sheer size but also with legitimate ethical and methodological limitations that do not allow all of the necessary datasets to be acquired directly from human material. This poses challenges for both empirical and digital research. Addressing such a challenge requires insights into the underlying structure of the brain, physiological phenomena in the organ, and a theoretical understanding of neural mechanisms.

Combinations of different methods, such as structural and functional magnetic resonance imaging (fMRI), magnetoencephalography (MEG), or electroencephalography (EEG), have successfully been applied to identify biological correlates of sensation, motor control, and executive function. However, closing the loops of understanding between cellular mechanisms and system-level effects requires multiscale neuroscience. Others emphasise that we also need to understand the “semantics” of how the various brain regions converse with each other (Douglas & Martin, 2007). As one example, according toBuzsáki (2019), global and local oscillations constitute the “syntax” for communication within the brain.

For many brain diseases, genetic mechanisms have been elucidated, with concrete relevance for diagnostics and therapy. Further, molecular and cellular mechanisms of several signal transduction pathways have been deciphered. Nevertheless, we are still lacking important insights into brain organisation, the relationship between brain structure, function, dynamics, and behaviour, its reorganisation during learning and sleep, as well as the conditions that underlie cognition. Simulation and the potential of AI to decipher the organisation of consciousness are already part of neuroscience discourse (see, e.g.,Dehaene et al., 2017;Graziano, 2019). The arrival of machines with capacity to simulate consciousness could mean that the “hard problem” of consciousness can be addressed by simulating the “easy problem” of consciousness (Chalmers, 1995).

While the multiscale architecture of the brain enables its resilience, adaptive capacity, and computational power, this property also significantly contributes to the inter-individual variability found at all levels of brain organisation. The degree of variability itself varies depending on the level, brain region and other factors (Croxson et al., 2018;Zilles & Amunts, 2013). Understanding variability will contribute to improved diagnostics and personalised therapies and will facilitate elucidation of the mechanisms of cognitive functions. In terms of basic science, this is a prerequisite for understanding both evolution and divergent cognitive profiles (Thiebaut de Schotten & Forkel, 2022).

Innovative neuroimaging, advances in microelectronics, and optical methods have opened a window onto brain function at ever-higher spatial and temporal resolution and over ever-longer periods of time, resulting in large amounts of data. Cohorts of thousands of participants have been enrolled with large numbers of data sets, but at lower resolution; these have facilitated the identification of factors determining brain health and aging such as lifestyle, environmental factors, genetic makeup, as well as the interplay between these variables. Such empirical research has resulted in significant volumes of highly structured data, a large amount of meta-data, and the increasing need for data integration.

So, what questions can already be answered based on the current data and where is additional work needed? Sydney Brenner stated during his 2002 Nobel lecture, “Nature’s Gift to Science” (Brenner, 2003): “We are drowning in a sea of data and starving for knowledge. The biological sciences have exploded, largely through our unprecedented power to accumulate descriptive facts … We need to turn data into knowledge, and we need a framework to do it”. Although a large amount of data exists, the research aims and methods used in individual laboratories are generally very diverse and data often cannot be directly compared with each other. Moreover, multi-dimensional data, with high quality, rigorous quality control, and provenance tracking (e.g., functional imaging data with simultaneously high spatial and temporal resolution and broad coverage including omics data), are sparse.

Such data do usually not come from one lab, but from many. Therefore, it has become clear that defining and achieving ambitious scientific goals will require close collaboration between laboratories with expertise in different areas of neuroscience and complementary technical expertise, for example, specialists in image analysis, neuroanatomy, data analysis, computation, physiology, biomedicine, modelling, theory, and computing. Several (neuro)ethical issues and questions regarding societal needs and value are relevant when studying the brain and brain diseases—recognition of this fact is leading to closer interaction between neuroscientists and researchers from humanities. Taken together, these developments enhance multidisciplinary collaboration, which needs to be appropriately organised and valued.

Such close collaboration across different domains of brain research is a defining feature of big international projects like the HBP^2^. The HBP is a European Flagship project in the field of Future and Emerging Technologies that started in 2013 and concluded in 2023. In 2013, the HBP was launched with the aim of achieving a deeper understanding of the brain, a goal that aligned with the remarkable advancements in computing and digital technologies during that time (Amunts et al., 2016,^2019^;Markram et al., 2011). The HBP was one of the first large-scale brain research projects worldwide and played a pioneering role in transforming digital brain research into a discipline that is more collaborative, reproducible, and ethically and socially responsible (Amunts et al., 2022).

The HBP has developed foundations for scientific workflows that enable a FAIR (findable, accessible, interoperable, and reusable;Wilkinson et al., 2016) comparison among multiscale, multi-species experimental data and theoretical and data-driven models (Eriksson et al., 2022;Schirner et al., 2022). To give a few examples, research in the project has led to new insights into the mechanisms of learning (Bellec et al., 2020;Cramer et al., 2020;Deperrois et al., 2022;Göltz et al., 2021;Jordan et al., 2021;Manninen et al., 2020;Masoli et al., 2021;Stöckl & Maass, 2021;van den Bosch et al., 2022), visuo-motor control (Abadía et al., 2021;Pearson et al., 2021), vision (Chen et al., 2020;Svanera et al., 2021;van Vugt et al., 2018), consciousness (Demertzi et al., 2019;Lee et al., 2022), sleep (Capone et al., 2019;Le Van Quyen et al., 2016;Rosanova et al., 2018), spatial navigation (Bicanski & Burgess, 2018;Northoff et al., 2020;Stoianov et al., 2018;van Beest et al., 2021), predictive coding and perception (Oude Lohuis et al., 2022), as well as language (Dehaene et al., 2015) and has resulted in new theoretical concepts and analysis methods. A special issue of the journal Neuron^3^was devoted to cognitive architectures in 2015. The aim was to bundle together research that is key for understanding and modelling human brain function, with many of the featured publications resulting from collaboration in the ramp-up phase of the HBP (Dehaene et al., 2015).

The neuroscience community has been empowered to take advantage of the most recent developments in computing, simulation, and artificial intelligence. Experimental data, computational models and tools, instruments, and dedicated hardware such as neuromorphic systems have been created in the project and made available with the intention of significantly speeding up developments in brain medicine and research as well as providing a model for low-energy consumption for the semiconductor industry (“Big data needs a hardware revolution,” 2018). The consortium has developed EBRAINS as a collaborative research platform with the aim of bringing brain research to the next level through digital tools and computation and of further developing applications in medicine and neuro-inspired technologies. EBRAINS is now part of the European Strategy Forum on Research Infrastructures (ESFRI) Roadmap. ESFRI aims to support a coherent and strategy-led approach to policy-making on research infrastructures in Europe and to facilitate multilateral initiatives leading to the better use and development of research infrastructures, at the EU and transcontinental levels. EBRAINS is being developed as a sustainable research infrastructure—by scientists for scientists.

To address ethical and societal questions, the HBP has incorporated principles and practices of Responsible Research and Innovation (RRI) into EBRAINS at the governance and research levels. The goal is to anticipate, reflect on, and undertake network-wide action on these and future neuroethical, philosophical, and societal and legal challenges and proactively address issues on dual-use research of concern, misuse, and commercialisation of EBRAINS research and its outcomes (Stahl et al., 2021). Looking to the next decade, we here identify gaps in our knowledge of the brain based on what has been achieved and articulate research goals for the future. We believe that efforts towards achieving these goals will benefit from progress in digital brain research as well as recent developments at the interface of technology and computing. Digital brain research takes advantage of fields such as data science, artificial intelligence, computing, modelling, and simulation, atlasing to enable progress in brain research, and to translate it into medicine and technology. These aims will also profit from the integration of neuroscience with neuroethics and multidisciplinary collaboration that engages with ethical and societal questions of need, acceptability, and desirability.

This manuscript has been developed in a participatory process (Annex 1). The work has been initiated by the HBP, and the entire research community was invited to contribute to shaping the vision by submitting comments. This more than 2-years process resulted in substantial changes of the original document, a broader representation of research concepts, sometimes controversially discussed, and a focused discussion, for example, with regard to the role of modeling and simulation. The authors converged in their formulation of common goals and steps to achieve them. While we do not claim that there is a “one-size-fits-all” approach to addressing these aspects, we are convinced that discussions around the theme of digital brain research will help drive progress in the broader field of neuroscience (seeAnnex 2).

## 2 Neuroscience: State of the Art

To understand what is missing and to motivate our approach for digital brain research, it is critical to consider where we have come from. To illustrate a few key steps on this path: modern neuroscience was born in the last two decades of the 19^th^century, when the brain, hitherto basically regarded as an unstructured mass, became recognised as an intricate network of individual cells, the neurons (DeFelipe, 2009;Mazzarello, 2010;Shepherd, 2015). New concepts on the segregation of the brain into areas, which are relevant for a certain function, gave rise to microstructural brain maps at the beginning of the 20^th^century (e.g.,Brodmann, 1909;Vogt & Vogt, 1919). Systematic neuropathological studies contributed to a deeper understanding of the brain both in health and disease. The full-brain electroencephalograms of the 1930s paved the way for intracellular electrophysiological recordings in the 1950s and to a basic understanding of the physiology of neurons and synapses. The discovery of the concept of chemical neurotransmission in the 1930s and the subsequent pharmacological revolution in the 1950s had great implications for neurology and psychiatry (Carlsson et al., 1957;Dale et al., 1936;Vogt, 1954) as well as for our basic understanding of how distributed computing networks like our brain can adapt flexibly to our changing world (Dayan, 2012). The Hodgkin–Huxley model was introduced in the 1950s to describe in mathematical terms action potentials (Hodgkin & Huxley, 1952). Explorations of the physiology of the sensory (mainly visual) and motor systems in the 1960s and 1970s, and parallel advances in their anatomy, provided valuable insights, giving rise to an updated view of the brain that we nevertheless now understand was somewhat naïve and simplistic (Shepherd, 2009). The 1980s saw great advances in our understanding of neuronal membrane biophysics and the functioning of receptors and ion channels (Sakmann & Neher, 1984), while in the 1990s the advent of full-brain imaging techniques kickstarted a period of intense progress in understanding brain organisation, its relation to genes and environment as well as individual variability. Novel techniques, including molecular biology, genetics, pharmacology, psychophysics, neuroimaging, and computational neuroscience, in combination with electronics and computing, have progressively enriched brain studies (Finger, 1994).

The beginning of the 21^st^century saw the development of new tools to manipulate and study brain circuits such as optogenetics, which, through activation or silencing, for the first time allowed investigation of the role of specific neuronal types (Deubner et al., 2019;Emiliani et al., 2022;Häusser, 2021;Südhof, 2017). Novel high-resolution imaging techniques, such as two-photon calcium imaging employed in animal experiments, have vastly improved our understanding of cellular and subcellular physiology (Toi et al., 2022;Yang & Yuste, 2017). In parallel with two-photon imaging, wide-field calcium imaging emerged as a powerful tool in systems neuroscience, allowing recording from multiple brain regions simultaneously with a sufficient spatio-temporal resolution to resolve behaviourally relevant information (Cardin et al., 2020;Ren & Komiyama, 2021b). The recent development of single-cell transcriptomics together with electrophysiological characterisation and morphological reconstructions have enabled researchers to obtain a solid basis of knowledge concerning the neuronal types in the mammalian brain (Chartrand et al., 2023;Fuzik et al., 2016;Gouwens et al., 2020;Lee et al., 2023).

It has been proposed that the global properties of stimuli could be encoded by neuronal synchronisation (Brama et al., 2015). For example, the “binding by synchrony” (Gray et al., 1989) theory held that features, like the colour and motion of visual objects, are consolidated into coherent perceptions when the neurons encoding these features fire at the same time, with millisecond precision. Later studies found that binding by synchrony does not occur (Lamme & Spekreijse, 1998;Roelfsema et al., 2004;Thiele & Stoner, 2003); rather, features of objects are bound into coherent entities by object-based attention which, at a neuronal level, increases neuronal firing rates (Poort et al., 2012;Roelfsema et al., 1998). Morphological and high-density recording tools for millisecond characterisation of brain circuits in animals carrying out specific tasks may be within reach in a few years for hippocampo-cortical networks (Klausberger & Somogyi, 2008;Lisman et al., 2017), motor cortex (Li et al., 2015), the barrel cortex (Staiger & Petersen, 2021), the basalo-cortical network (Gombkoto et al., 2021), and for some hypothalamic networks that organise sexual behaviours (Karigo et al., 2021).

At the same time, our theoretical and conceptual understanding of particular brain functions has also become richer and more complex. Links between anatomy and function can be investigated at various scales (Zaborszky, 2021). Microscale morphological features include myelo-, cyto-, receptor architecture, cell density, synapses, single neuron spike pattern, axonal and dendritic arborisation patterns, spine density, and gene expression, while physiological features range from ion channel biophysics to synaptic potentials or neuronal spike patterns. Studies have revealed area-specific synaptic organisation, receptor architecture, and arborisation patterns that show a surprising complexity of connections, though it is often unclear how these features contribute to specific processing differences within and between cortical layers and areal differences (Amunts et al., 2020;Haueis, 2021;Palomero-Gallagher & Zilles, 2019;Rockland, 2022).

At the macroscale, researchers, using MRI, describe the brain in terms of interconnected cortical areas, such as the macroscale connectional pattern that underlies hierarchical processing in the visual system (Felleman & Van Essen, 1991). At this scale, the brain exhibits spontaneous and systematic patterns of slow, low-frequency fluctuations in the blood oxygenation level-dependent (BOLD) signal measured in part in resting-state functional connectivity studies (Raichle et al., 2001). However, the precise relationships between BOLD imaging and details of electrophysiological patterns are yet to be determined. Architectural types are hypothesised to determine hierarchical processing (Barbas, 2015;Bastos et al., 2015;Mejias et al., 2016;Vezoli et al., 2021). The connectivity of transmodal areas allows them to integrate multiple unimodal sensory representations into categorical and rule-based areas (Mesulam, 1998;Pandya et al., 2015). Progress has been made in bridging connectivity between areas and the neuronal complexity of components within areas. Specifically, the functional imaging BOLD signal used in many human studies correlates best with local energy consumption (Viswanathan & Freeman, 2007), likely reflecting dendritic activity and interneurons mapped onto layer-spanning neurons and cortical layers. Such local microcircuit and dendritic activities serve important cognitive functions involving the comparison of internal models and top-down expectations with bottom-up information flow. These local computations might make a crucial contribution to the cellular mechanisms of conscious processing (Aru et al., 2020) and be missed in other electrical recording techniques measuring neuronal outputs. The understanding of layer-specific computation will be an important computational breakthrough that can be achieved by combining recording techniques sensitive to local microcircuit activity and dendritic activity (Larkum et al., 2018) with corresponding theoretical models of cortical computation (Haider et al., 2021;Sacramento et al., 2018).

The so-called mesoscale has been defined at the level of microcircuits, where researchers describe the brain in terms of different cell types and their connectivity and emergent dynamics. However, the relevant units remain a matter of debate. While in the 1970s, cortical columns of various sizes (minicolumns, hypercolumns, etc.) were thought to be functional modules (Jones, 1983;Mountcastle, 1997;Rockland, 2010;Szentágothai, 1978), continued discussions propose a combination of basic circuitry types, including feed-forward excitatory, recurrent feedback excitatory, feed-forward inhibitory, recurrent feedback inhibitory, and inhibitory–inhibitory types (Nadasdy et al., 2006). These circuits have been shaped through evolutionary pressure. Thus, it is important to understand the logic of evolving and maturing cortical circuits in order to identify specific circuits across species; this will tell us to what extent discrete anatomical features carry similar or dissimilar functions. An understanding of mesoscale circuits is important for properly linking micro- and macroscale descriptions of brain organisation, in order to properly infer macroscale behaviour from microscale features (Haueis, 2021). To this aim, wide-field fluorescence imaging can bridge the gap between neural activity at micro and macro spatial scales and provide understanding regarding how local circuits relate to larger neural networks (Cardin et al., 2020;Ren & Komiyama, 2021a). The limitations of individual techniques can be mitigated by combining different recording modalities (Allegra Mascaro et al., 2015); for example, recent studies used wide-field calcium imaging with other imaging methods, such as two-photon calcium imaging and fMRI (Barson et al., 2020;Lake et al., 2020). In order to rigorously map the complexity of meso-scale architecture, as well as its relation to (cross-scale) connectivity (Axer & Amunts, 2022), it is now possible to image molecularly defined cell types in the same (full) human brain section as cellular architecture (Kooijmans et al., 2020). Such an approach allows for a better understanding of how different cell types connect, at a local, as well as at a global level.

In parallel, a recent trend has been to focus on the geometry and dynamics of neural populations (Ebitz & Hayden, 2021;Saxena & Cunningham, 2019). One hypothesis motivating this approach is that (the most meaningful) neural activity takes place in low-dimensional state spaces or manifolds that capture a significant fraction of neural variability, and which can be identified by using dimensionality reduction techniques on high-dimensional neural recordings. Studying the geometry and dynamics of low-dimensional state spaces is suggesting novel mechanistic hypotheses about how the brain controls movements (Churchland et al., 2012) and how it supports perceptual and cognitive tasks (Chung & Abbott, 2021).

In order to connect the different scales and understand the rules of transition from one scale to the next, detailed models linking these spatial and temporal scales are necessary. In addition, biophysical models are needed that describe how physiological processes are captured by the measurement devices. For example, such models can be used to combine invasive electrophysiology that probes multi-unit activity and local field potentials of a neuronal population across cortical depths with high-resolution laminar fMRI (Havlicek et al., 2015): consisting of a microcircuit model including layer-specific distribution of excitatory and inhibitory neuronal subpopulations describing electrophysiology, which then provides the input to the fMRI signal model, and generative models of the fMRI signal consisting of models of neurovascular coupling, haemodynamic response, and physics of the BOLD signal.

The increasing understanding of this complexity in brain organisation went hand in hand with the rise of computational conceptualisation of mental phenomena and the success of artificial neural networks.Marr (1982)recognised that, in addition to the level of neural implementation, there are two further levels of organisation: the algorithmic and the computational levels. The need to involve computational neuroscience has grown in parallel with computational capabilities, which have expanded in the 21^st^century to the point where computational neuroscience has become an essential companion of both experimental and clinical studies. Apart from the modelling of concrete processes or computations, we can now consider more ambitious, larger, and integrative models. These models will inevitably shed light on the brain’s cognitive architecture and contribute to the development of more general artificial intelligence. Brain theories integrate the computational models within conceptual frameworks and formulate principles of their functioning grounded in information theoretical frameworks such as the Free Energy Principle (Friston et al., 2006;Parr et al., 2022) or dynamical systems theory such as Structured Flows on Manifolds (Jirsa & Sheheitli, 2022). In addition to modelling biological information processing, computational approaches enable large and complex data sets to be analysed efficiently, supported by the artificial neural networks, theory, modelling, and simulation, allowing the linking of brain structure and function. Simulation at cellular-molecular-level and/or in system models can facilitate the testing of specific hypotheses or prediction of properties of brain structures, dynamics, and even behaviour, while integrating findings from different researchers and obtained with various techniques. The integration of all experimental findings (models, texts, images, and other data) into a unified knowledge framework is still necessary. This, in turn, is critical for translating findings from neuroscience into digital medicine, for proposing new strategies of intervention and for empowering neuro-inspired technologies that take advantage of a growing body of insights into perception, plasticity, learning, and memory.

Current state-of-the-art technologies to study processes across the entire spatio-temporal spectrum are typically tailored to a specific species, genus, family, order, class, or phylum. Methods developed at different branches of the phylogenetic tree (e.g., invertebrates) are only slowly being adapted for usage at other levels, for example, rodents and primates. Recently, an annotated atlas of all cells and cell types has been released for Drosophila (Li et al., 2020), and genetic specification of circuit changes has been studied that results in functional changes at the macro level (Handler et al., 2019). This information may be important for understanding how macro-level state transitions may relate to individual differences in connectivity strengths (Taylor et al., 2022). Integrating this knowledge from model animals and translating it to humans by accounting for the effects of evolutionary diversification through statistical integration of phylogenetic knowledge (e.g.,Felsenstein, 1985; for an early mention of the need for this approach) would allow researchers to bridge scales in the human brain noninvasively.

Other examples of successful research in invertebrates are the exquisite reversible perturbation tools to dissect the functioning of micro- and macro-circuits (e.g., optogenetics, chemogenetics, pathway-selective perturbations), which were first developed in algae and further refined in invertebrates. These tools have gone on to revolutionise rodent research (Kim et al., 2017) but have only recently begun to be integrated in primate studies (Gerits et al., 2012;Han et al., 2009;Klink et al., 2021). Other species like zebrafish are being selectively employed to understand genetic or ontogenetic mechanisms that cannot be properly tested in mammals, for example (Rastegar & Strähle, 2016). Targeted perturbations can also be introduced by CRISPR/Cas9 into induced pluripotent stem cell models of neurons or brain organoids.

Currently, neuroscience references phylogeny (evolutionary history) when a trait is compared across two or more representative species. The identification of evolutionarily convergent traits in two distantly related species can be used to triangulate evidence of associations between related features (e.g., a brain structure and its associated behavioural function). The identification of evolutionarily divergent traits that differ between closely related species is used to pinpoint the origin of species-specific specialisations (e.g., a brain feature found in humans but not in other primates). In recent decades, genomic sequences for diverse species have formed the basis for an explosion of phylogenetic information, and with this has arisen a whole new statistical toolset for comparing traits across different species, called phylogenetic comparative methods.

Phylogenetic comparative methods have risen with the availability of digital datasets and the possibilities of comparative neuroimaging (Friedrich et al., 2021). They will certainly provide new opportunities to computationally analyse the ever-growing body of comparative neuroscientific data. They can provide statistical tests for inferences of homology; they can model how well a trait is conserved in evolution and they allow the convergence of traits to be examined quantitatively in a larger group of taxa. As more complex brain data become available in digital form and for more species, it will be possible to model the evolution of brain organisation, neural circuits, and cellular biology, along with genomic, epigenetic, and transcriptomic mechanisms. For example, structural brain connectomes have now been investigated in 125 mammalian species in comparison to phylogenetic distances (Faskowitz et al., 2022). In addition, new possibilities are arising through studies of ancient DNA, which have so far been used to connect human-specific features of gene expression to neuroanatomy by investigating Neanderthal contributions to human DNA (Gunz et al., 2019). Some of the alleles that are at present associated with human neuropsychiatric disorders might have previously been linked to these adaptations that arose when*Homo sapiens*—and the groups we recently admixed with—adapted to different environments around the world over time (Benton et al., 2021). As extant data and comparative fossil records about neuroanatomy, genomes, physiology, and behaviour continue to accumulate, new opportunities will continue to arise. Comparative data and evolutionary models could be used to develop AI by “reverse engineering” the minds of humans (Sendhoff et al., 2009), as well as other species, by documenting the changes that occurred during their natural histories.

Besides this evolutionary approach, neuroscientists study various model species at the systems level to understand specific principals of brain structure and function, aside from classic primate and rodent models. While there is much reliance on mouse models to understand the neurobiology of diseases and although mice are instrumental in tackling some diseases in humans, there are many human disorders for which they are not suitable models (Brenowitz & Zakon, 2015). For example, mice are commonly used to understand aging, but aged mice lack many of the biological features characteristic of human aging and diseases. Some model organisms do age in ways that resemble humans. Notably, cats and dogs recapitulate many aspects of human aging, and exhibit brain atrophy and cognitive decline with age (Gunn-Moore et al., 2007;Landsberg et al., 2012;Youssef et al., 2016). Neural pathologies in the brains of some cats and dogs share similarities with those observed in Alzheimer’s disease (Head et al., 2000,^2005^). Broadening the range of model systems used to understand human health and disease could help us address challenging problems in human medicine.

Although their brains are vastly different to those of mammals, avian models have become popular for investigating the fundamentals of complex cognition. This includes functions like memorisation of spatial routes or hundreds of food caches, problem-solving, social altruism, theory-of-mind, and multi-tasking (Balakhonov & Rose, 2017;Emery, 2006;Güntürkün & Bugnyar, 2016). Birds have outstanding cognitive capabilities, and songbirds possess a song system that is comparable to the human speech system. This means that birds are so far the only animal model for studying the development and processing of speech information in the brain, which has greatly stimulated research within the field of comparative neuroanatomy and pallial evolution (Brainard & Doupe, 2002;Brenowitz et al., 1997;Jarvis, 2004,^2019^;Nottebohm, 2005). Further, after more than 365 million years of separate evolution, birds have evolved a different pallial (neocortical) brain organisation compared to mammals but show similar connectivity between relevant brain areas, neurochemical features, neuron numbers, and gene expression profiles of cells that are functionally related to cognition (Colquitt et al., 2021;Herold et al., 2011,^2014^;Kverková et al., 2022;Shanahan et al., 2013;Ströckens et al., 2022). Such comparisons can yield basic insights into the links between brain structure and function and offer the unprecedented chance of gaining deep conceptual insights into fundamental brain functions. These studies could potentially identify a core of identical neural mechanisms in the brains of birds and mammals that constitute hard-to-replace components of advanced cognition (Stacho et al., 2020). Large-scale comparative research is key to understanding cognition and provides unique tools for deciphering the neuronal mechanisms underlying normal and pathological human brain functioning.

However, to what extent humans/primates evolved unique structural properties remains an open question. For example, the number and complexity of pyramidal cells, interneurons, and glial cells as well as specific brain network properties may vary between human and non-human mammals (Benavides-Piccione et al., 2020;Berg et al., 2021;Fang et al., 2022). Those studies included only a small selection of mammalian species, and it is not foreseeable if these differences will persist when additional species and/or parameters are considered. Furthermore, although previously thought to be unique to humans (Balsters et al., 2010), the neocerebellum likely expands predictably in all primates (Magielse et al., 2023). Methods have now been developed that allow us to examine human brain organisation and function at a level of detail close to what we can obtain with animal models (Eyal et al., 2018;Montero-Crespo et al., 2020).

Although by far not comprehensive, this overview of modern neuroscience illustrates several important points: (1) Advances in neuroscience are not only the result of conceptual advances but are tightly linked to new methods and technologies; (2) New techniques allow a better understanding of the brain, but at the same time open the door to a new level of complexity and open up new questions; (3) There is an increasing need for integration of knowledge and collaboration across different domains, scales, species, and models.

## 3 Instrumentation

Many new tools are facilitating profound insights into the brain’s structure and function; further, researchers also have at their disposal new capabilities and considerable computational power to analyse data and simulate brain function. Such tools are provided by different platforms and consortia worldwide.

We here focus on EBRAINS: a dedicated distributed digital research infrastructure for neuroscience. EBRAINS^4^gives access to data, tools, methods, and theories that were previously fragmented and distributed between different labs, in a joint, digital, open, interoperable platform. It has been developed in the HBP and operates according to FAIR data principles (Wilkinson et al., 2016). EBRAINS encompasses services for the sharing of neuroscience data and models, the multi-level atlas of the human, atlases of rodent and non-human primate brains, simulation, brain-inspired technologies, medical data analytics, as well as dedicated tools for collaboration. In addition, it incorporates innovative neuromorphic computing and allows for the execution of experiments in virtual robots. Fenix^5^, an infrastructure coordinated by experts from leading European centres for high-performance computing, greatly facilitates research with high computing and storage demands. Through Fenix, neuroscientists can also collaborate with other research communities to jointly develop new software and solutions in the broader domains of data- and computationally-intensive research. This is important because it creates synergies where different communities have similar questions (e.g., visualisation of large data sets, fast and interactive access to data), and it helps to use resources more efficiently.

The EBRAINS research infrastructure attracts a broad and very heterogeneous community of users, ranging from experienced application/service developers and senior neuroscientists to young researchers and students. Collaborative work and co-creation among stakeholders and users will be an essential part of the EBRAINS community and guide its development. The platform puts significant emphasis on the ease of use of its tools, and the interface complexity is balanced with user needs. This facilitates collaborative work, by combining tools to form computational workflows that seek solutions to diverse problems (e.g.,Eriksson et al., 2022;Fothergill et al., 2019;Wagner et al., 2022). In that sense, EBRAINS is changing the research paradigm scientists use to study the brain, both for large-scale neuroscience and for individual projects.

Computational workflows should be characterised by accessibility, shareability, automation, reproducibility, interoperability, portability, and openness. In this context, of particular importance is the use of the Knowledge Graph, ^6^ which includes a multi-modal information representation as well as the following “independence” features of EBRAINS workflows:

- Independence of tools and services from the workflows in which they are used. The inputs of tools and services are parameterised so that they may produce different outputs depending on other tools and services with which they are (re-)used in diverse workflows.
- Independence of workflows from the underlying infrastructure in which they are executed: the Common Workflow Language (CWL)^7^is being adopted for describing workflows in a common, standard fashion, offering transparent execution in infrastructures with different requirements, dependencies and configurations.
- Independence of workflows from the underlying workflow management system. Several such systems are compatible with CWL for executing workflow steps, monitoring their execution, handling failures, automatically fetching logs and outputs and other relevant actions.

This provides a technological basis for a new approach to international, collaborative neuroscience and represents a large-scale interface for collaborative projects, for example, organised in the International Brain Initiative (IBI)^8^and the NIH BRAIN Initiative (Litvina et al., 2019). Along the same lines, the European EBRA consortium developed a Shared European Research Agenda to increase the impact of brain research, advance basic, translational, and clinical brain research, improve the lives of persons with brain disorders, enable brain innovation, and address societal and economic challenges in Europe and globally^9^. Others have used the term Knowledge Representation (KR) to emphasise the need for a correct, robust, and verifiable representation of the vast neuroscience corpus (Di Maio, 2021).

To provide another example: recognising the importance of digital brain research and the potential benefits and value-driven impact for cognition, behaviour, and mental health, Malaysia has established the Malaysia Open Science Platform (MSOP)^10^as an initiative to strengthen science, technology, and innovation in Malaysia itself as well as outside the country’s borders. Going beyond the brain, on an even broader scale, the Human Reference Atlas (Borner et al., 2021) and the European Commission’s Virtual Human Twin (VHT) initiative (driven by the EDITH coordination and support action;https://www.edith-csa.eu/) aim to develop the necessary infrastructure to facilitate the creation of integrated multiscale multi-organ twins of the whole human body. Such twins may benefit from the lessons learned and the tools developed in EBRAINS.

## 4 What is Missing?

Deeper insights into brain function and dysfunction are not only now possible but are also urgently needed. Neurological and psychiatric diseases create a significant burden for those directly affected, carers, relatives, and society. Achieving progress in these areas is additionally motivated by philosophical questions of knowing and understanding our own nature, consciousness, and cognition. These different perspectives have to come together for a better understanding of the basis of brain health and the border between brain life and death. Ethical, philosophical, legal and regulatory, cultural and political challenges, which are intertwined, will need to be addressed concomitantly.

Progress in brain medicine is tightly linked to advances in basic research, but some fundamental questions remain open. To name a few examples, the formation of memories and the basis of conscious perception, the interplay of electrical and molecular-biochemical mechanisms of signal transduction at synapses, the role of glial cells in signal transduction and metabolism, the role of different brain states in the life-long reorganisation of the synaptic structure, the relationship between dynamical and cognitive models, or the mechanism of how cell assemblies generate a concrete cognitive function are all important aspects that remain to be characterised. Moreover, the specific, dynamic consequences of variations in brain organisation, including cyto-, myelo-, chemoarchitecture and interregional connectivity, are not yet well understood, but ultimately influence the local ratio of excitatory to inhibitory cell activity, resulting in a variable balance across different brain regions (Barbero-Castillo et al., 2021;Deco et al., 2018;Demirtaş et al., 2019;Jancke et al., 2022;Kringelbach et al., 2020).

Our current understanding of the mechanistic operations which subserve cognitive functions, such as memory or decision making, is limited by the scale and precision of existing technologies—simultaneous microscopic recordings are limited to a few brain regions, while full-brain imaging lacks the spatial and/or temporal resolution needed. Computational models, which could help to fill this gap, are likewise at an impasse: mechanistic models of cognitive functions focus almost exclusively on microscopic scales (Amit & Brunel, 1997;Mante et al., 2013;Wang, 2002), while full-brain models are largely oriented to replicating large-scale neural dynamics (Breakspear, 2017;Deco et al., 2011). Novel modeling approaches must be developed to close this schism in the field, either by introducing simplified cognitive functionalities in large-scale brain models (Mejías & Wang, 2022), by extending cognitive models such as recurrent neural networks to multi-region frameworks (Yang & Molano-Mazón, 2021), or by increasing the biological plausibility of existing cognitive multi-region models (Dora et al., 2021).

The need for interaction with the brain (both “reading” and stimulation/manipulation), originally driven by clinical requirements, has opened novel and expanding fields such as the assessment of awareness in disorders of consciousness (e.g., unresponsive wakefulness syndrome, locked-in syndromes), brain-machine interfaces, cognitive enhancement, sensory restoration, and sense-expanding technologies, which have relevance beyond the medical sector for society at large. There is also a need for brain recordings of high temporal and spatial resolution and activity control that are at the same time minimally or non-invasive. These technological advances require interdisciplinary work from neuroscience and areas such as micro- and nanoelectronics, optics, light-controlled drugs, nanorobotics, new materials (e.g., graphene), etc. It is to be anticipated that advances in security, biocompatibility, reactive changes in the brain (e.g., gliosis, cell death), signal-to-noise ratio, problems related to invasiveness (surgical, infections), and closed-loop control of brain function will be made soon; these advances will bring with them consequences in terms of legal and ethical issues.

While progress in these fields has been impressive, a comprehensive understanding of underlying processes requires an integration of each system (e.g., visual, sensorimotor) with the rest of the brain, with the body, and with the environment. Furthermore, it requires integration of molecular, subcellular, cellular, and systems levels, to reach a “multiscale” understanding that incorporates the emergent properties of all these complex relationships. These levels cannot be fully understood by considering only parts of the system. Each level, when it malfunctions, may result in a large variety of neurological and neuropsychiatric diseases. In order to understand the process holistically, one needs to understand all the individual steps, which is today in many cases difficult or impossible. It is necessary to approach the individual steps at the relevant level of abstraction and to develop a theory, and, in addition, to have access to the relevant data at the different levels of brain organisation through a multi-level structural and functional atlas.

The newest computational bottom-up models are now able to integrate microscopic features, such as those of specific ion channels, synaptic receptors, and neuromodulators, and evaluate their impact at the level of cellular subpopulations. Recently, this approach was even extended to the whole-brain level, by studying the effect of molecular targets of anaesthetics, such as propofol, and their impact at the level of large-scale activity. For example, changing K^+^conductance (Dalla Porta et al., 2023), or the kinetics of inhibitory (GABA-A) synaptic receptors, can induce a switch of brain activity to synchronised slow-waves, similar to the effect of anaesthetics^11^. This is an example of an area where computational models can contribute.

A full causal understanding of how behaviour and cognition are produced through cortical computation requires the combination of both bottom-up and top-down approaches. The paradigmatic example is the ventral visual stream. While deep neural networks for object recognition have been inspired by the architecture of the visual system, these networks also provide an improved functional model of the visual system itself. In fact, the statistical properties of model neurons in the deep networks are closest to those of real neurons recorded in the brain (Yamins & DiCarlo, 2016;Zhuang et al., 2021). It remains a challenge to reproduce this functionality of the top-down models with more detailed bottom-up models.

This type of interplay between experimental measurements and modelling predictions is very powerful and has led to impressive advances in understanding network-level phenomena such as oscillations, waves (Breakspear, 2017;Marder et al., 2022;Tort-Colet et al., 2021). The extension of such an approach to the level of the whole brain, however, is more challenging because of the high level of complexity involved, as well as the still-insufficient temporal and spatial resolution of non-invasive human imaging and recording techniques. Linking these models with imaging requires a deep biophysical understanding of the different signals involved. This is particularly relevant when computational models are used to quantitatively predict cognitive function and aging (Charvet, 2021;Charvet et al., 2022;Heckner et al., 2023;Jonsson et al., 2019), for example, based on imaging data of patients and healthy subjects and for building precise loops between computational models and clinical data, which should ultimately lead to a better understanding of neurological diseases.

Network and other models are also tools to investigate how physiological mechanisms can be perverted in pathological conditions, for example, where microscopic changes down to modifications at the protein level can lead to aberrant behaviour or clinical symptoms (Mäki-Marttunen et al., 2019). Among the best understood cases are epilepsy disorders, where several microscopic targets have been identified, leading to abnormally high excitability. Another example comes from a multifactorial causal model that included neurotransmitter receptor data and enabled the prediction of variance in the clinical severity of Alzheimer’s disease symptoms, thus further supporting the value of creating personalised brain models, as well as the importance of their enrichment with data arising from multiple modalities (Khan et al., 2022). In contrast, the tissue pathologies and brain signals of many other pathologies such as schizophrenia are not well understood, and computational models may have an important role in identifying mechanisms and also in predicting potentially informative macroscopic and/or behavioural features. To answer these and other research questions, a number of technological, methodical, and computational challenges has to be adressed (Box 1).

Box 1. Technological, methodical, and computational challenges.

Brain research poses enormous technological and computational challenges for brain interfacing, analysis, and mechanistic understanding, data interpretation, and modelling of brain processing. To cite but some examples:

- The complexity of data (multi-level brain organization, hierarchies, parallel information processing, redundancy, electrochemical processing, etc.). A key aspect of this complexity is the relationship between different scales that speaks to the level of granularity (and accompanying data) that is most apt for elucidating these relationships. One approach from physics is the notion of “renormalisation”; namely, the conservation of laws from one scale to the next (sparse coupling, hierarchical dynamics, computational principles, etc.). In addition, measurements at all relevant scales are required to obtain information on how low-level states combine to generate states at higher levels, and to account for neurodegeneracy, that is, the propensity for different system configurations to support the same or similar functions.
- The multitude of data formats and data models arising from the use of diverse hardware, software, and analytical approaches. Data sourced from various researchers and laboratories often display disparities, creating hurdles for integration and interoperability. Promoting the adoption of standards and harmonisation procedures, including the utilisation of standardised brain atlases for spatial referencing, is essential. These measures play a pivotal role in facilitating data reuse and the combination and utilisation of data across different contexts.
- Brain data derived from human subjects can undergo de-identification but may not achieve anonymisation (rendered impossible to trace back to the individual). Consequently, there is a demand for secure data storage services that offer controlled or restricted access to facilitate data reuse. In these protected storage systems, making data discoverable involves openly sharing anonymous metadata, a practice currently employed by EBRAINS.
- Many behaviours and some mechanisms are unique to humans, but a large proportion of data is not directly accessible and remains unknown (e.g., chemical reaction kinetics at the cellular level cannot be measured in the living human brain). Comparative approaches studying animal brains as well as modelling and simulation are strategies to overcome this problem.
- Intersubject variability and diversity. It is necessary to integrate information from diverse human populations for personalised medicine into atlases, databases, and research.
- The specific spatial and temporal resolution of data sets, given the multiscale nature of brain spatial and temporal activity. Scale integration is challenging (from micro- and nanometre scales, through meso- to macroscale) as is the capture of brain dynamics. This requires representation of different scales in a common framework according to the topography of the findings, that is, in multi-level and multiscale atlases and models that account for the temporal domain.
- The large size of “subsystems” (e.g., large molecules such as neurotransmitter receptors with many atoms and complex, dynamic structures, large networks, whole-brain perspective as compared to regions of interest, large cohorts).
- The wide spectrum of response patterns, dynamics, plasticity, and behaviour of the system in pathological conditions.
- The changing nature of the system, which manifests plasticity at different spatial scales (from dendritic spines to large networks; processes such as spike adaptation, long-term potentiation, long-term depression) or neurodegeneration after lesions.
- The accuracy and reliability of predictions and analyses, applicable to individual subjects, which is particularly critical for translating applications into brain medicine.
- The lack of a comprehensive brain theory, or a selection of competing theories.
- The lack of integrability and documentation of extensive brain collections using modern experimental approaches, including those over 100 years old in Europe and worldwide, to make better use of historical brain preparations and data. These number in the many tens and hundreds of thousands of specimens and, for the most part, are not yet digitised and/or available via web-based tools. Some of them include rare species or brains obtained under conditions that cannot be reproduced any more (e.g., untreated patients with brain disorders). Making this digitally accessible for researchers worldwide would be of significant benefit to evolutionary, comparative, and also clinical research; however, this aspiration is linked to significant challenges in data exchange, storage, and security. First attempts are underway to combine post-mortem brain dissections with*in vivo*imaging in a digital framework, for example,https://bradipho.eu/.

## 5 Ethical and Societal Questions as Drivers of Responsible Digital Brain Research

Digital brain research should be driven by scientific curiosity and a desire to promote society’s best interests; further, it should reflect societal priorities, including a better understanding of the brain, the development of better diagnostic tools, and more effective treatment of brain diseases. In this section, we briefly suggest how we can ensure that societal concerns are addressed and reflected in the research and its outcomes and describe approaches for guaranteeing that research and innovation processes are carried out responsibly. Future research programmes must integrate anticipatory practices, neuroethical reflection, multi-stakeholder and citizen engagement and support ongoing compliance with current legislation, regulation, and good research practice. This includes careful consideration of the role of gender and diversity in data generation and governance of research, attention to potential dual-use research of concern or misuse of neuroscientific findings, as well as reflection on the ethical sustainability of the research, its impact on human rights, and its long-term societal and political implications. Additional social and legal issues to be considered in relation to digital brain research include those raised by data protection and the European Commission’s General Data Protection Regulation-compliant data governance (GDPR), social desirability, acceptability, and sustainability of digital brain models and issues raised by the possibility of advanced artificial cognition, brain-inspired computing, and neurorobotics research, among others. In one example, the intersection of neuroscience and technology is likely to lead to new approaches to AI. In digital brain research, the emphasis should not only be on amassing vast amounts of data but also on ensuring a diverse representation, encompassing factors such as sex, age, and ethnicity. This inclusivity extends to researchers, practitioners, and stakeholders involved. By embracing diversity, the field can effectively address issues related to biases in AI and proactively engage with emerging concerns arising from innovative approaches, technologies, and applications.

The framework of Responsible Research and Innovation (RRI) defines a multidisciplinary approach to tackling the ethical, philosophical, societal, and regulatory challenges that accompany the vision of future digital brain research. Furthermore, RRI-inspired research and practices can be useful in building a future where responsible digital brain research is proactive in its recognition of existing and emerging societal and ethical challenges.

Digital brain models are a key concept and model for future brain research. They raise significant philosophical questions (e.g., what are the limits of access of brain–machine interfaces to other brains?) (Evers & Sigman, 2013) and ethical and social issues (e.g., are there potentially problematic applications of the technology? Who is involved in the analysis and decisions on potential applications? How would we like to use such models in society?) (Evers & Salles, 2021). Conceptual clarity is a prerequisite for informed debates on the ethical issues raised by digital brain research. Approaching such questions through the framework of RRI includes reflection on the meaning and adequacy of the concepts involved, engagement and dialogue between different disciplines in neuroscience research, including philosophers, ethicists, and social scientists with societal stakeholders like policymakers, interest organisations, and the public (seeBox 2).

## 6 Globalisation of Brain Research

The proliferation of digital technologies in brain research has expanded since the dawn of the 21^st^century and analysing multi-modal data from many thousands of brains, made openly available through public repositories (e.g., UK Biobank) or global networks (e.g., ENIGMA, HCP), is possible. Of course, access to dizzying amounts of data means nothing without the means to convert these data into knowledge and, ultimately, into a better understanding of the brain’s complex machinery in normal behaviour, in development or aging, and in brain disease. Accordingly, we have seen the rise of complex generative models that track the spatiotemporal progression of brain states (Iturria-Medina et al., 2018;Vogel et al., 2021;Young et al., 2018) by combining genetic and phenotypic information across multiple time points. AI strategies are playing an increasingly important role in classifying massive cohort data into rationally defined sub-groups that may be amenable to customised interpretation, for example, polygenic risk scores of behavioural predisposition or stratification of pharmaceutical clinical trials. Finally, such approaches offer the potential for personalised management or medical intervention.

However, the search for ever more subtle and early biomarkers of incipient changes in brain state often demands ever larger aggregates of data to tease out the factors that are associated with, or perhaps cause, those changes. This search brings with it the perennial conflict of homogeneity versus representation. While there is little doubt that “big data” approaches applied to large public data repositories, for example, ADNI, PPMI, UK Biobank, etc., have provided us with hitherto unmatched insight into the general nature of the human brain’s mechanisms and circuits, such cohorts are largely drawn from Western countries and are not representative of the global population.

The effectiveness of data repositories requires sufficiently rich and diverse data to ensure that outcomes of research and the innovations informed by these outcomes can be generalisable to diverse populations and contexts globally. Sex differences, age, socioeconomic status, ethnicity, and other factors contribute to individual differences in neural structure, function, and cognitive performance (Dotson & Duarte, 2020) as well as differences in disease prevalence, recovery, and survival rates between demographic groups (Sterling et al., 2022;Zahodne et al., 2015). Moreover, differences worldwide exist regarding the reporting of racial demographic information in studies (Goldfarb & Brown, 2022). At the same time, initiatives in Low- and Middle-Income Countries (LMICs) have steadily grown for the diagnosis and prevalence of brain disorders and mental health issues, for example, the ASEAN region. There is a need for global collaboration, including the collection, dissemination, and analysis of well curated, deeply phenotyped, and genotyped datasets from LMICs to identify similarities and differences among different global sub-populations. It is not possible to obtain statistically reliable inference about such comparisons without access to nationally representative cohorts from different countries, a requirement beyond the reach of individual laboratories. As the repeated use of existing datasets leads to their inevitable decay (Thompson et al., 2020), the problem of representation cannot be addressed merely as an afterthought but requires urgent prioritisation.

In the coming decade, as open data-sharing initiatives (UK Biobank, OpenNeuro, CONP, EBRAINS, etc.) expand globally, scientists' evolving views on data management and sharing (Donaldson & Koepke, 2022), along with shifting expectations from funders and journals (see, e.g., Editorial in Nature Neuroscience (“How we promote data sharing,” 2023)), will likely result in a significantly increased availability of diverse data for the global community. This will bring a new level of awareness of the associated and causal factors that give rise to brain and behavioural differences among global populations. Such data-sharing platforms, many of which have now been in existence for over a decade, have reached a level of technical advancement such that they already support open data-sharing across many countries.

However, there is work to be done in developing a clear and seamless interoperability across diverse platforms, ensuring that end-users can engage without delving into intricate technical underpinnings. The challenge is not merely about providing “data”; the emphasis lies on delivering data that are both valuable and interpretable, complete with provenance that adheres to FAIR data-sharing principles (Wilkinson et al., 2016). Technically, achieving data interoperability, providing data descriptors and protocols, and adhering to metadata standards not only enhance the value and usefulness of the data but also contribute to building a stronger, collaborative, and more efficient research ecosystem. However, the imperative for access to meaningful and actionable data also introduces a myriad of challenges related to data governance and ethics. These practices are still evolving across different constituencies, with diverse and sometimes incompatible frameworks globally (Eke et al., 2022). Differences also exist regarding the reporting of racial demographic information in studies (Goldfarb & Brown, 2022), and the technical capacity to generate and process data, funding for data collection, and other socio-cultural factors. So far, datasets from regions in Africa and Latin America are often not part of global brain research and innovation discourse.

The next decade will see a pressure to harmonise the different data governance and ethics frameworks in Europe (e.g., GDPR), North America, Asia, Australia, and Africa, to foster the wider dissemination of brain data within an Open Neuroscience global community. More attention should be paid to capacity building, increased reporting of demographic information, funding programs, and finally awareness campaigns focused on data generation, processing, and sharing in low- and middle-income countries.

Arguably the most important aspect of the globalisation of brain research will be the “democratisation” of brain research. Rather than being simply sources of cohort data that are analysed and published by scientists in High-Income Countries, we anticipate a growing presence of LMIC scientists in the brain research enterprise. This democratisation is a natural evolution from the increasing access to advanced analytic workflows that are available through current data analytic portals (e.g., CBRAIN (https://cbrain.ca/), EBRAINS (https://ebrains.eu/), BrainLife (https://brainlife.io/). Such portals allow researchers anywhere in the world to run complex analyses on large datasets that are resident elsewhere and remove the logistical, administrative, and technical barriers that have hindered LMIC scientists from participating fully in the brain research community. Further, the redistribution of derived data becomes possible by combining data sharing and analysis platforms. The sharing of results is essential to minimise scientific redundancy, maximise reproducibility, and foster accessibility of scientific analyses to LMIC environments. With growing awareness of the role, that analytic decisions play in learned models of the brain (Botvinik-Nezer et al., 2020), the dissemination of derived data allows for both iterative and collaborative approaches to scientific exploration and removes key barriers to entry. Such a vision also brings with it a host of administrative factors to be worked through, for example, academic recognition, promotion, mentorship, etc., but these issues are already topics within the current Open Neuroscience debate. Adding a globalisation component introduces scaling and logistical challenges, for example, language, local governance regulations, but does not change the fundamental issue, which is the tension between data privacy and open science. We anticipate that, as the technical challenges are resolved, the vision of global neuroscience integration will become a reality over the next 10 years.

## 7 Brain Models as Enablers of Future Brain Research

The accelerated development of information and communication technologies in the past two decades has not only supported the development of simulation and machine-learning technologies but has also made data and models interoperable within a common ecosystem leading to novel types of brain models. Directly tapping into the results stemming from basic research on the brain, brain simulation is expected to play a key role in elucidating essential aspects of brain processes (by demonstrating the capacity to reproduce them*in silico*), such as decision-making, sensorimotor integration, memory formation, etc. While mindful of some of the ethical and philosophical issues they raise, one may also envision the potential use of such models and simulations to address specific questions in brain research. From there, it is easy to envision how generic brain models can be customised to capture some of the distinct features of a given patient’s brain. For example, an individual’s structural and functional brain imaging data may constrain a generic digital brain model and render it subject-specific, thus enabling its use as a personalised analysis template or*in silico*simulation platform.

A concrete instance of such an approach is the*Virtual Epileptic Patient*, wherein neuroimaging data inform*in silico*simulations of an epileptic patient’s brain to support diagnostic and therapeutic interventions, clinical decision-making, and prediction of consequences (El Houssaini et al., 2020;Jirsa et al., 2017;Wendling, 2008). With the overall trend in computational neuroscience, various models of epileptic activity are being built based on knowledge regarding the relevant underlying neural circuits. The models often explain the network-level observation of epileptic seizures as an emergent hyper-synchronous/high amplitude rhythmic state of network of neurons or neural population. Multi-level atlas data represent another data source that can further inform personalised brain models in instances where data cannot be directly obtained from that subject (Amunts et al., 2022).

Such personalised “virtual brains” can be seen as a stepping-stone towards something even more theoretically and technically, and possibly ethically challenging, but also better adapted to the ever-changing nature of brain activity across all time scales. The logical culmination of personalised brain simulation can be seen in a model that is continuously informed and updated by real-world data, a type of model referred to as a “digital twin”. The concept of the “digital twin” in this context needs to be carefully defined to avoid obscuring the limitations of the approach and to avoid creating unrealistic expectations of exact fidelity or even counterproductive hype (Evers & Salles, 2021). Historically, the concept of the digital twin originated in the realm of industry and manufacturing (Grieves & Vickers, 2017;Grieves, 2019), and comprises three components: the physical object, its virtual counterpart, and the data flow back and forth between the two. Empirical data measured for the physical object are passed to the model, and information and processes from the model are passed to the physical object. Today, the term “digital twin” is widely used beyond its origins in the industrial domain and is now applied in many areas of research, including in biological and medical fields, although the concepts behind this term may differ.

In manufacturing, the digital twin is more than a general simulation model. It is the specific instance of the general model for an individual object fed with empirical data from that specific object, for example, an airplane engine in the industrial domain (Tao et al., 2019). Recently and in the same context, “digital shadows” have been proposed as an improved approach to provide task- and context-dependent, purpose-driven, aggregated, and persistent datasets that can encompass different complex realities from multiple perspectives in a more versatile fashion and with better performance than a fully integrated digital twin (Becker et al., 2021;Brauner et al., 2022).

One reading of a digital twin speaks to the dialectic between machine learning and generative modelling in AI. Generative models underwrite interpretability and explainability. Furthermore, they enable the move from “big data” to “smart data” (or more precisely selecting and integrating data features to maximise expected information gain). A generative model is a probabilistic specification of the mapping from (latent) causes to (measurable) consequences. In this sense, a digital twin can be taken as a formal specification of a model that is apt for generating the responses of a cell, subject or cohort in question. Crucially, getting the generative model right affords an interpretable and mechanistic account of empirical data. Coincidentally, it casts the distinction between bottom-up and top-down modelling in terms of model fitting (i.e., inversion) and model selection (i.e., hypothesis), respectively.

In constructing a “digital twin” of a living organ, one is confronted by important challenges over and above those encountered when constructing the digital twin of an inanimate object. The brain is by far the most complex and multi-facetted organ. To what extent, then, can the digital twin concept be applied to neuroscience and the brain? The term digital twin, if applied 1:1 to the brain, could trigger major misunderstandings. Here, we want to contribute to the discussion by clearly defining the term in the specific context of brain science. We distinguish purpose-driven digital twins from the abstract idea of a full digital replica (or duplicate/copy) of the brain, the latter being the complete representation of all aspects of the brain at all levels (seeBox 3). A full replica of the brain is neither achievable nor does it seem of clear practical use. When we speak of digital twins in what follows, we mean purpose-driven digital models generated for specific questions, unless explicitly indicated otherwise. The digital twin as discussed here should be understood as a virtual model designed to adequately represent an object or process that is constrained by data from its physical counterpart and that provides simulation data to guide choices and anticipate their consequences. The digital twin is thus a copy in the practical sense, usually associated with a model of a function or process, and its power lies in its usefulness in dealing with relevant problems faced by its physical counterpart at an appropriate level of abstraction. The aim is thus not to resemble the biological brain in as much detail and on as many levels as possible but rather to selectively reduce the amount of information to those data that have proven predictive for a specific (research) question—keeping the model as simple as possible but ensuring it is as complex as necessary.

Box 2. Ethical questions.

Acknowledgement of ethical questions that arise as a consequence of digital brain research, especially by digital twins.

- Privacy. Digital twins are constantly updated with real-world data. These data can be identifying, particularly when imaging, genetic, and clinical data are combined. Even “siloed” sources of information, in great enough quantity, can prove identifying, especially in cases of rare diseases. Increasingly, it appears that promising de-identification may not be possible in the face of big data (Choudhury et al., 2014). It is crucial that individuals be informed of privacy considerations during the consent process and that they understand that the identification risk may increase over time (White et al., 2022). As a community, and in collaboration with governing agencies, policies will need to be established regarding these aspects in the future.
- “Mind-reading”. Concerns about privacy are amplified given that much of digital brain research investigates emotion, perception, memory, and mental states: realms that are often considered sacrosanct aspects of inner life. Already, brain imaging (alongside various physiological measurements) has been shown to be predictive of behaviour at the population level (Bell et al., 2019;Talozzi et al., 2023). Digital brain models have the potential to be even more powerful: for instance, they may suggest how to enhance particular brain states, in addition to merely classifying them (Ligthart et al., 2021) .
- Malfeasance. It is increasingly recognised that digital brain research can be “dual use”. It may equally cause harm and bring benefits.

Even for a specialised model that aims to understand specific aspects of brain structure and dynamics or predict the progression of disease in a specific patient, one still needs a comprehensive source of data to draw from in order to generate sufficiently information-rich, complex Virtual Brain models. Such curated data systems have been created, for example, in the form of the Human Brain Project’s high-resolution multi-level human brain atlas on EBRAINS. These serve as an interface for integration of structural and functional data modalities. With each model, it must be demonstrated whether more data makes the model more powerful or not, that is, do the added data enable more accurate, testable predictions? There needs to be a continuous, question-related monitoring of the trade-off between the inclusion of more parameters or measurements for better predictions and the feasibility and associated costs of collecting these data. This also serves as an ongoing loop for testing whether the data selection is suitable for the question at hand, that is, whether it reflects the major determining factors (Box 3).

Box 3. Categories of digital brain models.

- **Brain models**
  Brain models are digital representations of the brain. The term is used in different contexts; common examples include digital atlases, artificial neural networks, anatomical models, biophysical models, network models, cognitive and behavioural models, and mathematical and data-driven models.
- **Personalised brain models**
  Personalised brain models are special types of models that are personalised by integrating specific data of one individual into a more general model (e.g., as enabled by the Virtual Epileptic Patient).
- **Digital twins**
  Next-generation personalised brain models that continuously evolve by being informed with real-world data. They are designed in a purpose-driven way, integrating data relevant for a specific research question.
- **Full replica**
  The idea of a complete digital representation of all aspects of a brain at all levels (hypothetical concept), eventually including the interpretation with the digital twin body

An important distinction between the digital twin and other personalised virtual brain models is that the digital twin constantly receives new information from the real world to immediately adjust to its environment. In a neuroscience context, a “digital twin” of a brain in the above sense holds much promise as an approach for continuously adapting interventions in functional neurorehabilitation or for tailoring neurotechnology-based interventions. Applications making use of a high-fidelity digital twin of a human brain updated in quasi-real time will require technical developments (e.g., ecological immersion of that twin brain in simulated environments, high-bandwidth, stable brain-machine interfaces, very high computational power), in areas where breakthroughs have yet to be made; as such, they remain a long-term objective for a rather distant future. This is not to say, however, that digital twins cannot already be applied in neuroscience and medicine today, provided they adequately address the intrinsic limitations of current brain models, of available personalisation processes and those faced by current technologies in updating them at the required frequency. The twin thus defines the current horizon of our digital neuroscience roadmap and must be appropriately taken into account as a driver for future developments.

While the use of digital twins of the brain in concrete applications may still seem some way off, the era of digital brain research has, without question, already started, both in real world settings and research alike. Digital brain research is an umbrella concept under which data, models, theory, methods, and computational technology are integrated for all research and development efforts undertaken in the framework of the HBP. Its value rests upon a successful demonstration of internal and external validity (features of experimental results) as well as ecological and construct validity (features of interpretative claims). It enables researchers to address some of the major challenges that have hindered progress in neuroscience for decades. These challenges include our understanding of intra- and inter-subject variability, non-identifiability of mechanisms, and multiscale complexity. EBRAINS provides an infrastructure and user interfaces to allow interoperation of the required components of data, models, and methods; in doing so, it*de facto*establishes the operational basis for the concept of the digital brain to take centre stage in neuroscience research.

We propose that there are three areas where digital brain models of all kinds (seeBox 3) could be fruitfully applied in the short-to-medium term: (1) basic brain research, (2) applications in medicine, and (3) brain-derived technologies.

### 7.1 Basic brain research

Digital brain models and their simulation will not replace basic research and knowledge accumulation but can be rather thought of as a useful “engineering” tool that functions currently as an in-progress predictive model with a dual purpose: (1) putting current knowledge to the test, and (2) anticipating the effect of interventions. The latter can be appealing as the number of interventional methods is expanding (deep brain stimulation (DBS), transcranial magnetic stimulation (TMS), transcranial direct current stimulation (tDCS), transcranial focused ultrasound stimulation (tFUS), drugs, optogenetics, and photopharmacology). Although there are already various studies where computational brain models make predictions, drive the design of and explain effects observed in interventional research (Frank et al., 2004,^2007^), these methods are currently often applied “semi-empirically” with the available information about electrode location; circuit connectivity, function, and electrical models; genetic promoters of neuronal types; expression patterns of neuroreceptors and their signalling pathway models, etc. The digital twin may allow rational decision-making regarding these parameters, the testing of outcomes, followed by re-evaluation of the model and so forth.

In order to be successful, underlying models must be biologically realistic, that is, anatomically adequate and functionally comprehensive. Ultimately, they should be capable of linking brain structure and function with behaviour and allow the study of cognition, language, consciousness, or emotions. This requires the integration of highly heterogeneous data across scales, including*in vivo*and*ex vivo*, in the same spatial reference framework. In an alternative, complementary approach, the Cell Atlas Network (BICAN) will extend to the whole human brain the approach used in the US Cell Census Network (BICCN), undertaking in-depth characterisation of (small-scale) components of the mammalian brain, for example, the most detailed and comprehensive multi-modal model of the primary motor cortex including single-cell transcriptomes and proteomes, chromatin accessibility, DNA methylomes, spatially resolved single-cell transcriptomes, morphological and electrophysiological properties, and cellular resolution input-output mapping (Callaway et al., 2021).

Based on this concept, brain simulation plays a key role in elucidating brain complexity by allowing the testing of hypotheses about the brain’s multi-level organisation and its functions that control the surrounding body (see also next paragraph). Clearly, following this line of research, it will become more and more important to interconnect simulations executed at different spatial levels (e.g., the EBRAINS simulation engines Gromacs at the molecular level, Arbor and NEURON at the cellular level, NEST at the systems level, The Virtual Brain at the whole-brain level, and the neurorobotics platform at the level of the embodied organism and its environment (see*Brain-derived technologies*)); for an overview seeEinevoll et al. (2019).

Unlike with the real living brain, the embedded simulated brain can be sampled at any point in space and time. It will be possible to look at all the processes in such a brain (provided those processes are modelled in the simulation, based on real-world data and/or on physics/chemistry) and make this observation with simulated measurement devices, for example, multi-array electrodes, fMRI scanners. Then, in principle, all kinds of functional hypotheses can be tested in a full-body and closed-loop environment; further, it will also be possible to build dynamic anatomical atlases, for example, atlases that allow for the observation of the changes and processes in a brain section under a specific stimulus—in real simulation time.

The multiscale complexity of the living brain, the limited accessibility for measurements, and our incomplete understanding of brain processes make the realisation of the digital twin approach difficult to say the least. The BigBrain as an anatomical model may serve as the scaffold for the integration of twin data in a strict sense (Amunts et al., 2013), data from other sources such as dynamic cellular data and those from experimental population studies, as well as synthetic data simulated by models and different brains. Such an approach also determines the limitations and ranges of validity of the digital twin strategy, which is crucial for the responsible use of and subsequent trust in the technology. Nevertheless, such data-driven models may represent the closest digital representation of a living human brain that is achievable at any given point in time. New insights from mathematics will be necessary to speed up simulations and analyse models (Lehtimäki et al., 2017,^2019^,^2020^).

Therefrom, the following goals can be derived:

○ Develop multi-level brain atlas and high-resolution brain models.
○ Enable multi-level brain models and simulation.
○ Elucidate the mechanisms of cognition and behaviour.

### 7.2 Brain medicine

From such digital twins, personalised twins can be derived with the aim of improving diagnostics and therapy for patients in a new and powerful way and therefore supporting strategies towards brain health such as that recently published by the European Academy of Neurology (Bassetti, 2022). Analogous to cardiac digital twins (Gillette et al., 2021), that is, digital replicas of patient hearts derived from clinical data that match all available clinical observations, human electrophysiological replicas have great potential for informing clinical decision-making and also for facilitating the cost-effective, safe, and ethical testing of novel device therapies. Digital twins in medicine address a defined spatial scale, with a defined granularity, consider a defined time interval, and serve a dedicated purpose. An application of the digital twin approach for Alzheimer’s disease has been proposed recently (Stefanovski et al., 2021), and while careful consideration of data privacy, security, and safety aspects will be required, personalised twins might also offer a uniquely powerful strategy for treating such conditions.

The Virtual BigBrain (TVB) enables construction of individual connectomes based on neuroimaging and EEG data of a subject and anatomical data from the BigBrain model (Jirsa et al., 2017). The ongoing*EPINOV*clinical trial employing the TVB represents a major step forward in this regard; scientists have developed individual models of the brains of patients undergoing epilepsy surgery to guide and predict the best seizure outcome (Jirsa et al., 2023;Proix et al., 2017;Wang et al., 2023). Here again, the strategy is to combine population data with data from an individual brain to develop a Virtual Brain model, a twin, that is realistic enough to allow simulation of the intervention prior to surgery. Patients with super-refractory seizures, that is, seizures which persist over periods of anaesthesia, often require prolonged intensive care and are at a very high risk of permanent neurological damage and death. For such patients, a digital twin might be used to examine a vast array of models, with ongoing feedback from EEG, responses to drugs and blood ion and gas concentrations, all readily available in intensive care environments.

The utility of digital brain modelling is illustrated by DBS, a well-established surgical therapy for several treatment refractory neurological disorders. Currently, clinical-use DBS most often implements an “open-loop” system, meaning that stimulation is delivered continuously according to fixed parameters. These parameters can be adjusted after implantation, but adjustments are manual, infrequent, and driven by observation of patients’ visible symptoms. In contrast, “closed-loop”, adaptive DBS has been developed to overcome limitations of traditional DBS and to modulate neuronal circuits based on clinically relevant biofeedback signals in real-time (Marceglia et al., 2021). To apply them successfully, however, requires understanding mechanisms of plasticity and learning.

Applications following localised brain lesions, such as stroke or traumatic brain injury, would have similar requirements. Beyond invasive therapeutic interventions, a digital twin would be a powerful tool for predicting the consequences of brain lesions, pathophysiology, and plasticity, which is sometimes described in terms of computational neuropsychology, namely, characterising lesion-deficit relationships*in silico*, using synthetic lesions (Parr et al., 2018). This could significantly change our capacity to personalise neurorehabilitation, while integrating complex information generated by virtual reality and robot-based therapies together with fine measurements of patients’ responses and progress.

Other applications could employ simulations to test a “clinical” simulated population that could be far larger than a real one, therefore providing data amplification by creating cohorts of “digital patients”. This could be particularly interesting for evaluating rare diseases, for studying the influence of gender, or for predicting disease progression (Maestú et al., 2021). Moreover, the more diverse (and heterogeneous) the sources of data used for training, the better the performance of the model on other datasets, resulting in good generalisability. This is one of the most interesting features provided by federated systems, which facilitate increasing the diversity of data sources (e.g.,Dayan et al., 2021).

Recently, the AlphaFold system developed by DeepMind (Jumper et al., 2021), an application of deep learning methods, has enabled prediction of protein 3D structure. This could be generalised to test the drug-protein or drug-protein-system interactions at a systems level. Another perspective would evolve from testing the effect of drugs in a virtual environment to uncover the mechanisms of the drug not only at molecular but also systemic levels. Considering that quantum mechanics/molecular mechanics are computationally highly demanding, such an approach at a systems level would require highly scalable tools run on the most powerful supercomputers. For example, fine-grained models of local microcircuits with molecular or cellular resolution, like those constructed and simulated using NEURON and Arbor, can be directly used to map the local distribution of some molecules (e.g., ion channels, receptors) and then be used to simulate the impact of drugs on this system. These low-scale models can be tuned according to a given pathological condition and then transformed into patient-specific mean field models, advancing the precision of digital twins.

More generally, increased cross-talk between the neuroscience fields addressing the human brain as compared to those focusing on non-human brains could work synergistically to solve long-standing problems in biomedical sciences (Devinsky et al., 2018). Humans and companion animals suffer from overlapping diseases (e.g., epilepsy, cancer, obesity). Similar to humans, dogs suffer from epilepsy and are subjected to brain scans when they are sick. The overlap in diseases and care offered by human and veterinary medicine means that there are untapped opportunities to test the effectiveness of personalised medicine and digital twins in companion animals before implementing them in humans.

Finally, it would be expected that brain twins contribute to “human body twins”. This perspective goes beyond merely adding another organ, because it would allow modelling the interactions of nervous system activity with those of other organs at the systems level, for example, heart-brain couplings and linking the brain with stomach and intestines. These interactions are pervasive and bidirectional. For example, recent research has identified an intrinsic allostatic and interoceptive system in the human brain, which includes visceromotor regions that provide cortical control of the body’s internal milieu and support allostasis (Kleckner et al., 2017). Furthermore, bodily processes such as respiration are powerful drivers of rhythmic neural activity (Tort et al., 2018). Capturing these bidirectional interactions would help us understand how the brain supports vital bodily functions—and possibly how to restore them when they are impaired.

The challenge of bidirectionally and systemically linking multiple single-organ or single-scale digital twins is a key element of the European Commission’s roadmap for the Virtual Human Twin that is currently under development (https://www.edith-csa.eu/).

Therefrom, the following goals can be derived:

○ Obtain detailed insights into brain plasticity, learning, adaption, during the lifespan.
○ Accelerate digital brain medicine.
○ Explore and model the brain as part of the body.

### 7.3 Brain-derived technologies

A fundamental challenge is to establish what level of granularity in brain modelling, what transitional computations, and what kind of simulated development are required to support the emergence of a variety of cognitive and sensorimotor functions. Models of the human brain, simulated in embodied settings, that is, having the ability to control virtual or physical bodies interacting with realistic virtual or actual physical environments, and receiving time-dependent input streams to produce behavioural outputs, represent a uniquely attractive platform for investigating the links between brain structure, brain activity, and cognitive and functional performance.

How such bottom-up assembly and the emergent behaviour of the digital twin system can be evaluated against biological data remains an ongoing challenge, because typical synthetic development environments do not match the natural environment.Yong (2019)argued in his feature article “The Human Brain Project Hasn’t Lived Up to Its Promise” in The Atlantic^12^that “large-scale simulations are useful for understanding weather and galaxies, but “planetary systems are not about anything other than themselves. A brain is built to be about other things.”…. Simulating the tissue is do-able, but meaningless.”

The previous paragraphs provide several examples where simulation has led to progress in basic neuroscience and brain medicine for well-defined research questions. Additionally, the HBP from its start aimed to develop technologies enabling the study of brain-environment interactions (“Booklet | Brain-inspired intelligent robotics: The intersection of robotics and neuroscience sciences,” 2016). In other words: a simulation of certain processes occurring in the brain is embedded in a real or simulated body with all its sensors and actuators connected to the simulation. In principle, these sensors and actuators can just as well be real or simulated or a combination thereof. Likewise, this body is embedded in a real or virtual world. Once we have these elements, simulated or real, we can combine them in any sensible way.

Obviously, this approach is heavily dependent on models representing the physics of the real world, and it also requires sophisticated software that can simulate spatial environments in high fidelity and that can provide adequate physics of environments, sensors, and actuators, connection to brain simulators, facilities for storing the results of simulations, graphical rendering, and the orchestration of these complex software modules. All of these (co-)simulations can be run at different time scales (ideally of course in real-time), in closed-loop or open-loop scenarios, and with entities modelled at different granularities.

The neurorobotics platform of the HBP^13^is a software environment that was designed to perform all these steps, run simulations based on diverse sets of data from biological experiments as well as input from real world robots, and integrate machine learning on top of those simulations. While this platform was originally conceived of for the purpose of designing neurorobots, that is, robots that are controlled by biologically inspired models of the brain, over time it has evolved into a software environment that can be used to connect and integrate all types of entities ranging from simulated mouse bodies by way of sophisticated sensor models to various neuron and brain simulators. Today, the neurorobotics platform can be considered to be both an environment for robot design, and at the same time, an execution platform for neuroscientific experiments. It is therefore a powerful vehicle for*virtualising neuroscience*, up to the point where system-level*in vivo*experiments can be replaced with*in silico*experiments that run completely inside this platform.

In addition, the neurorobotics platform allows for training the “brain” (AI-based controller) of embodied robots with real neuroscientific data, even before they are built. It is also conceivable that a simulated copy of the real environment in which they will be used serves as the reference basis for the training, so that they can be pre-trained before they are shipped to the end user, who will only need to make small adaptations to (emergent) behaviour to ensure that the robot performs its tasks in a perfect manner. We will refer to approaches following this paradigm as brain-derived technologies, as they are directly based and built on findings from brain research. Importantly, these findings can be implemented at different levels of organisation. In neuromorphic engineering, the main components, that is, biological neurons, are emulated by functionally equivalent electrical circuitry to construct highly energy-efficient, possibly analogue, processors, and sensors. Likewise, the neural models running on these systems can be derived from specific types of neurons, microcircuits, or brain regions that have been identified in biological brains. When connecting these systems to robotic embodiments (both simulated and/or physical) or to biological organisms, it becomes possible to replicate some aspects of the full closed loop of perception, cognition, and action. Modelling can thereby be extended to the complete organism and address all aspects of complex cognitive processes at the behavioural level. Brain-derived technologies are therefore not limited to approaches that mimic structural features of the brain but can also encompass cognitive models and architectures along with their underlying neural dynamics. These technologies will represent new tools for brain research and enable innovations in computing, robotics, and AI.

One field expected to benefit greatly from this approach is neurorehabilitation, where realistic models of brain-body interactions will be useful in elucidating the neural mechanisms at play (Rowald & Amft, 2022). The combination of highly detailed brain models with models of the spinal cord and of the musculoskeletal system indeed affords special opportunities, such as allowing investigation of the relationship between neural activity and resulting motor behaviour in a detailed, quantitative manner. Personalised models could thus be integrated into decision-support systems to guide the choice and combination of rehabilitation strategies by a physician or a therapist. They may also support breakthrough developments in central nervous system (including spinal cord) stimulation technology and functional electrical stimulation, improving the efficacy of these techniques and expanding their relevance to a greater breadth of conditions. A very promising recent application reported successful epidural electrical stimulation to treat spinal cord injury (Rowald et al., 2022).

Similarly, the combination of high-fidelity models of both the human musculoskeletal and central nervous systems is also expected to support the emergence of*in silico*technologies for so-called*electroceuticals*, that is, medical devices that provide neurostimulation for therapeutic purposes (e.g., in Parkinson’s disease, epilepsy, etc.). There is little doubt that the medical device industry would have a fundamental interest in tools guiding their product design, generating predictions regarding efficacy, and overall de-risking of the whole product development process. With the brain atlases and the multiscale brain simulators created by the HBP, it thus seems timely to consider the collection and integration of new data (e.g., dielectric properties) as a prelude to the development of simulation tools and services geared towards the evaluation of electroceuticals. Simulating the effect of such electroceuticals seems to be overdue, given that DBS is already being widely used.

The HBP has supported the SpiNNaker many-core and BrainScaleS physical emulation neuromorphic computing platforms in establishing the first open neuromorphic computing services and has contributed to the further development of these technologies (Furber & Bogdan, 2020). Neuromorphic technologies, where both data transfer and processing are event, that is, spike-based, provide a multitude of opportunities for edge computing, mobile robotics, and neuroprosthetics. Considering current trends in automation of mobile systems and deployment of “always-on” sensor arrays, in particular, neuromorphic devices are expected to deliver enhanced, low-latency capacities for perception, cognition, and action, while reducing the impact of onboard operations on the system’s energy consumption (Cramer et al., 2022;Göltz et al., 2021). For example, combining spike-driven processing units with spike-generating sensors (e.g., dynamic vision sensors, dynamic audio sensors) into complete neuromorphic systems (sensors and processing units) will make it easier to perform data fusion and overcome constraints related to the heterogeneity of data sources. Advances in the neurocomputational understanding of learning by neuronal circuits, especially through synaptic plasticity, will also provide new ways of endowing neuromorphic circuits with ever-more complex functionalities at a lower training cost (e.g., one-shot and continuous on-line learning). In particular, the restriction to local plasticity constitutes a manifest advantage over conventional von Neumann architectures.

The circuitry of analogue neuromorphic processing systems such as BrainScaleS emulates the ion flows in biological neurons by electrical currents. Unlike traditional microprocessors that are based on the classic von Neumann architecture, every silicon neuron is physically incorporated into the chip with dedicated components. Like in the brain, these neurons exchange information based on spikes, which allows for an extremely efficient implementation and is one of the reasons why neuromorphic systems are a promising technology for a new generation of real-time-capable and extremely energy-efficient computers. An important consequence of their direct derivation from the brain’s structure is that neuromorphic processors are typically not well suited for loading external data but instead support learning online in real-time. This unique feature enables new types of learning rules that do not require large data sets but adapt dynamically as required.

Learning rules based on spike timing-dependent plasticity are a remarkable success story of brain-derived systems (Diamond et al., 2019;Kreutzer et al., 2022). They are directly rooted in experimental results and have become a cornerstone for research on learning algorithms in both theoretical neuroscience and neuromorphic engineering. Importantly, traditional machines have also benefited considerably from brain research. One of the most prominent examples are arguably convolutional neuronal networks, precursors of which have originally been derived from the architecture of the visual cortex.

Another important area where basic brain research has fostered the emergence of new technologies is that of neuromorphic sensors, particularly dynamic vision sensors and dynamic audio sensors. The former mimic the functioning of the retina and, like neuromorphic processors, encode information with spikes. The characteristics of these are completely different from their traditional counterparts. Since they only signal changes rather than capturing full image frames, they can operate extremely efficiently, give rise to new types of image processing algorithms, and ideally complement neuromorphic processors.

From a technological perspective, the human brain is also the most promising “Rosetta Stone” for the implementation of advanced cognitive abilities in artificial systems. Modern artificial agents are characterised by limited levels of intelligence, difficulty in generalising beyond provided training sets, and an often-superficial understanding of their environment. The lack of generalisability implies either the necessity for large data sets (the resource-intensive big data paradigm), continuous human supervision (remotely controlled systems), or extensive, rigid mission planners accounting for any allowable occurrence (for planetary or ocean exploration). The superficiality of perception and lack of explainability imply a lack of robustness of and trust in artificial perception systems, a known obstacle to the emergence of, for example, effective driving automation. To mitigate against such limitations, brain-inspired multi-area model architectures must be developed in conjunction with new embodied and incremental learning algorithms, with a view to finding those that best emulate the functional mechanisms underlying human perceptual cognition. Harnessing such mechanisms and understanding the emergence of cognitive functions will be essential for creating explainable, reliable, and eventually more general AI.

The functional architecture of the brain with its different regions is the basis for many types of cognitive architectures that have been defined for technical systems. This is especially true for robotics, where brain-derived approaches are studied extensively. Examples include the research on phenomena related to embodiment or the development of novel perception and sensing systems such as artificial whiskers, inspired by the actual somatosensory system in rodents.

Future developments in neural networks for artificial intelligence applications will see a convergence between mainstream AI and neuromorphic technologies. Multiscale brain models can make a critical contribution to the construction of advanced robotic controllers. These could embed plastic rules and autonomously adapt through their interaction with the environment. Thus, basic brain science will be key in informing the development of these technologies. Moreover, neuromorphic computing might help reduce the substantial carbon footprint of large deep learning models (Strubell et al., 2019).

Therefrom, the following goals can be derived:

○ Bridge the gap between human and machine intelligence.
○ Build neuromorphic brain models and bio-inspired artificial intelligence.

## 8 Conclusion

An improved understanding of brain function depends on a deeper understanding of brain organisation and a better appreciation of the fundamental mechanisms—the actual biological processes, their relationships, and the rules that govern them. This is prerequisite to more efficiently target prevention, therapies, and mechanism-based diagnoses. A promising approach for the coming decade of digital brain research consists in developing digital twins of individual brains that afford personalised simulations. Although now feasible, digital twins of the brain are still at an early stage and once developed have to undergo rigorous testing and validation before they can meaningfully address brain disorders and become the basis for disruptive new health technologies. Therefore, we need to understand the computational goals and algorithms of the systems and subsystems to be able to see the limitations and possibilities of implementation in individual cases. Further, brain twins raise ethical questions that we will need to address in an open dialogue with society. Twins can be seen as a kind of endpoint for ongoing developments of brain models and analytics.

With this goal in mind, a digital infrastructure that can host such digital brain twins may foster progress in understanding the rules and refining our digital brain twins to a point where they pass validation testing and become useful for clinical translation. Further, such an infrastructure should ideally provide interoperability, information security, multi-level data, access to knowledge-based computing resources, including high-performance computing and other relevant technologies. EBRAINS is an infrastructure that is capable of hosting such developments. To make that successful, training of younger generations in working with such infrastructures and leveraging the potential of new digital tools is key.

Structuring data and knowledge such that they can easily be recombined and integrated towards a plethora of digital brain twins by the research community—together with delivering the powerful technology with which complex simulations of these twins can be performed—may in itself represent a disruptive technology for generating scientific insight.

## 9 Scientific Goals—a Roadmap

The “roadmap” below outlines goals within eight intersecting areas of research in the coming decade, each ranging from (1) near-term or current work, (2) middle-term, to (3) long-term. It is derived from the input provided above.

Develop multi-level brain atlas and high-resolution brain models

1. Integrate data, from the whole-brain level to cells, into a comprehensive, high-resolution brain atlas as a basis to get a deeper understanding of general principles of brain organisation, to enable the prediction of missing features, where the atlas is incomplete, and to guide comparative studies about interspecies similarities and differences.
2. Generate detailed, data-driven, multiscale models to study the role of variability in human brain organisation during lifespan, under different conditions.
3. Elucidate those aspects of brain organisation and structure that are responsible for complex behaviors, intelligence, and consciousness.

Enable multi-level brain models and simulation

1. Multiscale integration of models, from local biophysical properties to whole-brain models, including detailed bottom-up and top-down models. Models are driven and tuned by data and their predictions tested.
2. Model biologically realistic, complex brain functions using multi-scale, whole-brain models—approaching digital brain twins for concrete use cases.
3. Apply model predictions to larger-scale use cases in basic science, medicine, and AI, which, in turn, drive model testing and sophistication (“productive loop”).

Elucidate the mechanisms of cognition and behaviour

1. Develop a coherent framework describing the mechanisms of cognitive functions using a multiscale perspective, from sensory- and visuomotor to more complex cognitive functions.
2. Formulate a coherent framework for language, as a uniquely human complex cognitive function, integrating insights from linguistics and neuroscientific research using multi-level brain approaches, using development as a window to brain specialisation, and providing the backbone for development of language models and artificial intelligence.
3. Link concepts of different hypotheses and self-consciousness to each other and to mechanisms at the cellular, molecular, and genetic levels.

Obtain detailed insights into brain plasticity, learning, and adaption, during lifespan

1. Identify and integrate the rules of plasticity, learning, and adaptation, into existing multi-level brain models.
2. Identify constraints of brain plasticity, and tools to modulate it for the benefit of patients.
3. Reveal mechanisms of memory consolidation and translate this to medicine and technology.

Accelerate digital brain medicine

1. Develop and apply personalised models, informed by brain atlases and individual patient data, for diagnosis and treatment of a broad range of brain disorders (e.g., epilepsy, tumours, movement disorders, stroke, psychiatric disorders).
2. Construct and apply data-driven models of development and aging to brain medicine in different age groups (from children to the elderly).
3. Develop and apply digital body twins, continually amenable to new real-life sensor data, to brain medicine (e.g., diagnostics, rehabilitation, intensive care, and surgery).

Explore and model the brain as part of the body

1. Link advanced digital brain models to spinal cord models based on multi-level atlases and derive therefrom new approaches for stimulation.
2. Model sensorimotor integration and coordination for interaction, task performance, and navigation.
3. Integrate somatic and autonomic regulation in combined, multi-organ models to construct*patient twins,*which reflect nervous system, organ, and body regulatory functions. Develop and apply cellular-level body twins, which model nervous system, endocrine/hormone, immune regulatory, and homeostatic mechanisms.

Bridge the gap between human and machine intelligence

1. Simulate complex behaviour using robots interacting with rich environments; promote convergence of deep learning AI and event-based (spiking) neural networks facilitated by neuromorphic technology; democratise and develop complex (brain-inspired) AI models, including large language models in an open, transparent approach.
2. Apply insights into brain mechanisms behind cognitive functions, such as perception and decision-making, to emulate learning and developmental processes in the fields of AI and neuromorphic technology and test the potential role of organoids and organoid intelligence (OI).
3. Apply fundamentally new concepts and algorithms to machine learning and novel engineering applications (e.g., new materials, artificial life, replacing and enhancing brain function).

Neuromorphic brain models and bio-inspired artificial intelligence

1. Develop training methods for spike-based deep neural networks using leaky-integrate-and-fire-based neuron models. Integrate complex hardware neuron models in simulation environments.
2. Develop hardware and training methods for large-scale and highly performant spiking network models using complex neuron models.
3. Integrate results from plasticity research to develop large-scale spiking networks with built-in learning capabilities.

## Supporters

The following individuals express their support of the contents of this manuscript: Pietro Avanzini, Marc Beyer, Maria Del Vecchio, Jitka Annen, Maurizio Mattia, Steven Laureys, Rosanne Edelenbosch, Rafael Yuste, Jean-Pierre Changeux, Linda Richards, Hye Weon Jessica Kim, Chrysoula Samara, Luis Miguel González de la Garza, Nikoleta Petalidou, Vasudha Kulkarni, Cesar David Rincon, Isabella O’Shea, Munira Tamim Electricwala, Bernd Carsten Stahl, Bahar Hazal Yalcinkaya, Meysam Hashemi, Carola Sales Carbonell, Marcel Carrère, Anthony Randal McIntosh, Hiba Sheheitli, Abolfazl Ziaeemehr, Martin Breyton, Giovanna Ramos Queda, Anirudh NIhalani Vattikonda, Gyorgy Buzsaki, George Ogoh, William Knight, Torbjørn V Ness, Michiel van der Vlag, Marcello Massimini, Thomas Nowontny, Alex Upton, Yaseen Jakhura, Ahmet Nihat Simsek, Michael Hopkins, Addolorata Marasco, Shamim Patel, Jakub Fil, Diego Molinari, Susana Bueno, Lia Domide, Cosimo Lupo, Mu-ming Poo, George Paxinos, and Huifang Wang.

## Data and Code Availability

No additional datasets or code are associated with this paper.

## Author Contributions

All authors have contributed to writing, reviewing, and editing of the manuscript; the progress on how the paper has evolved can be found on Zenodo^14^. The conceptualisation of the paper has been initiated by the Science and Infrastructure Board of the Human Brain Project.

## Declaration of Competing Interest

The authors declare the absence of any commercial or financial relationships that could be construed as a potential conflict of interest.

## Footnotes

1
https://www.thelancet.com/gbd/about

2
https://www.humanbrainproject.eu/en/

3
https://www.cell.com/neuron/issue?pii=S0896-6273%2814%29X0043-7

4 EBRAINS:https://ebrains.eu/

5 Fenix:https://fenix-ri.eu/

6
https://search.kg.ebrains.eu/

7
https://www.commonwl.org/

8 International Brain Initiative:https://www.internationalbraininitiative.org/

9
https://www.ebra.eu/sebra/

10
https://www.akademisains.gov.my/mosp/

11 work in progress in showcase 3 of the HBP:https://www.humanbrainproject.eu/en/follow-hbp/news/2022/06/20/how-ebrains-used-investigate-disorders-consciousness/

12
https://www.theatlantic.com/science/archive/2019/07/ten-years-human-brain-project-simulation-markram-ted-talk/594493/

13
https://www.neurorobotics.net/

14
https://zenodo.org/records/10035197

## Annex 1

White paper: Participatory process and timeline

## Annex 2

Statements of support

Rafael Yuste: “As a European working in the US, I strongly support this initiative, it could help put European neuroscience in a leadership position and help European countries capitalize on the benefits of working together towards the same goal.”

Linda Richards: “Overall, this manuscript presents novel ways of moving the field forward and is extremely exciting.”

Alexandra A. de Sousa: “As founder of the European Network for Brain Evolution Research I strongly support this initiative and in particular its reference to the importance of comparative and evolutionary neuroscience.”

Mu-ming Poo: “Understanding the structure and function of the human brain and developing effective approaches in diagnosis and intervention of brain disorders are both long-term goals of all societies. The tasks are enormous, requiring global collaboration in promoting rapid progress and sharing knowledge and technology. China Brain Project is now fully funded by the Chinese government for the coming decade. Chinese scientists, many of them have close ties with scientists in the Europe and US, are hoping to establish international collaborative projects, and to set up effective mechanisms to facilitate collaboration.”

George Paxinos: “It is exciting to observe the progress that has been made in the development of multi-level brain atlases. The advanced digital tools that have emerged in recent years offer entirely new possibilities for studying brain structure in different species.”
